# Editorial: Adrenal neuroendocrine system and cardiometabolic health: pathophysiology and clinical implications

**DOI:** 10.3389/fendo.2023.1295655

**Published:** 2023-10-27

**Authors:** Moe Thuzar, Fady Hannah-Shmouni, Michael Stowasser

**Affiliations:** ^1^ Endocrine Hypertension Research Centre, The Frazer Institute, Faculty of Medicine, The University of Queensland, Brisbane, QLD, Australia; ^2^ Department of Endocrinology & Diabetes, Princess Alexandra Hospital, Brisbane, QLD, Australia; ^3^ Division of Endocrinology, University of British Columbia, Vancouver, BC, Canada; ^4^ Hypertension Unit, Princess Alexandra Hospital & Greenslopes Hospital, Brisbane, QLD, Australia

**Keywords:** adrenal, glucocorticoid, cortisol, mineralocorticoid, aldosterone, metabolism, metabolic syndrome, cardiovascular

Metabolic syndrome (MetS) and cardiovascular diseases (CVDs) represent the largest disease burden at a global level (1). The situation warrants a broader and more thorough understanding of the pathophysiological factors regulating cardiometabolic health to identify potential novel targets and/or guide effective screening and management of at-risk individuals. The adrenal neuroendocrine system plays a fundamental role in the regulation of energy and substrate metabolism, fluid and electrolyte balance, cardiovascular tissue remodelling and immune function, thereby underscoring cardiometabolic health. The articles in this Research Topic offer important insights into the implications of the adrenal neuroendocrine system, particularly the glucocorticoid (GC) and the mineralocorticoid (MC) systems, in the pathophysiology of cardiometabolic diseases.

GCs are adrenal steroids widely used pharmacologically for various inflammatory and autoimmune conditions due to their potent anti-inflammatory effects. Obesity, hypertension and impaired glucose tolerance are common metabolic complications of GCs. The paper by Deng et al. in the current Research Topic reported the results of a systematic review and meta-analysis of 43 studies evaluating the cardiovascular risk of GC treatment in more than 15 million subjects. They found that the major adverse cardiovascular events (MACE) risk increased by 10% for each additional gram of GCs cumulative dose or by 63% for an additional ten microgram daily dose. These findings have significant clinical implications as GCs are one of the most frequently prescribed therapeutic agents with millions of people taking GCs worldwide (2), and call for more rationalised prescriptions of GCs and for more research into identifying potential targeted agents to counteract the adverse cardiometabolic effects in people requiring GCs.

The MC system is emerging as a potential key player in the manifestations of cardiometabolic diseases, not just hypertension. Aldosterone is classically recognised as a main regulator of blood pressure (BP) via its effect on renal tubules promoting sodium and fluid retention. There is a soaring level of evidence linking overactivity of the MC system to features of cardiometabolic syndrome, including hyperglycaemia, adiposity, metabolic syndrome associated fatty liver disease (MAFLD) and cardiac dysfunction (3, 4). The paper by Wang et al. in this Research Topic highlighted the association between aldosterone and obstructive sleep apnoea (OSA). The paper by Gan et al., after examining more than 3000 hypertensive patients with suspected OSA (UROSHA data), found that those with plasma aldosterone concentration (PAC) in the third tertile had almost 2 fold higher risk of incident CVD and all-cause mortality, compared with those with PAC in the first tertile (HR = 1.81). The relationship was found in those with suppressed renin as well as in those without suppressed renin, indicating that having PAC in the high normal range despite not necessarily having primary aldosteronism (PA) is a risk factor for CVD, and the authors determined a threshold PAC of ≥12.5 ng/dL (≥350 pmol/L) to identify at risk individuals. These cardiometabolic comorbidities are likely mediated by the activation of mineralocorticoid receptors (MRs) in non-epithelial tissues such as adipose tissue, immune cells and cardiovascular tissue. Activation of MR by aldosterone or upregulation of MR suppresses brown adipose tissue (BAT) function, promotes adiposity and adipose tissue inflammation and cardiac remodelling and fibrosis (3–5). On the other hand, MR antagonism or MR deletion increases the activity of BAT, induces of adipose tissue browning, ameliorates adipose tissue inflammation, and protects against cardiometabolic dysfunction (3, 6, 7). The preclinical and clinical evidence together strongly suggests a causal relationship between the MC system and cardiometabolic comorbidities, and raises a number of questions; (i) is it time that MC overactivity be viewed as a risk factor for MetS and CVDs and be managed more holistically to reduce the cardiometabolic comorbidities, and (ii) should PA be considered and screened for as a potential underlying issue in patients with significant cardiometabolic diseases, ie., adopting a MetS-based screening and management approach for PA rather than the current BP-focused (BP-centric) approach?

Sexual dimorphism and potential interplays between the GC and MC systems in the pathophysiology of cardiometabolic manifestations are other areas receiving increasing interest. The paper by Ouyang et al. in this Research Topic investigated sex differences in hypercortisolism and glucose metabolism disturbances in autonomous hypercortisolism (ACS), and observed that the association between ACS and markers of glucose metabolism was more pronounced in women. The authors postulated that the findings might be due to binding of progesterone and estrogen to the GC receptor, the ligand-binding domain of which demonstrates 55% sequence identity with the progesterone receptor and 30% with the estrogen receptor. Sex differences in cardiometabolic risk has been well described, albeit with females in general having lower risk, attributable to the protective effect of oestrogen. The finding by Ouyang et al. of an apparent loss of the cardiometabolic protective effect of estrogen in the setting of GC excess is an interesting phenomenon which has also been reported in women with obesity; blood pressure is less likely to be adequately controlled (despite more likely to be treated) in obese women than men, thought to be mediated by the renin-angiotensin-aldosterone system (RAAS) (8). The paper by Chaves et al. in this Research Topic investigated the effect of the RAAS, particularly the angiotension-2 type 2 receptor (AT_2_ receptor) on the exacerbation of adrenal GC steroidogenesis in diabetic mice, and found that blockade of AT2 receptor by Olmesartan reduced GC production. The interplays between the GC, MC and other systems warrant further delineation to facilitate personalised targeted treatment approach for various at-risk groups.

In summary, there is little doubt that the adrenal neuroendocrine system plays a pivotal role in the regulation of cardiometabolic health, and the papers included in this Topic represent important incremental steps towards identification of novel targets and effective management of MetS and CVDs in at risk individuals.

## Author contributions

MT: Conceptualization, Writing – original draft, Writing – review & editing. FH-S: Conceptualization, Writing – review & editing. MS: Conceptualization, Writing – review & editing.
